# Measurement of microRNA with isothermal DNA amplification on fully automated immunoassay analyzers

**DOI:** 10.1007/s00216-019-01878-z

**Published:** 2019-06-03

**Authors:** Makoto Komori, Ken Komiya, Takuma Shirakawa, Takamitsu J. Morikawa, Toru Yoshimura

**Affiliations:** 1 Research and Development, Abbott Japan Co. Ltd, 278 Matsuhidai, Matsudo, Chiba 270-2214 Japan; 2 School of Computing, Tokyo Institute of Technology, J2-51, 4259, Nagatsuta-cho, Midori-ku, Yokohama, Kanagawa 226-8503 Japan

**Keywords:** MicroRNA measurement, Isothermal DNA amplification, Automated immunoassay analyzer, Cancer diagnostic marker, Chemiluminescence microparticle assay

## Abstract

MicroRNAs (miRNAs) in a blood sample are usually measured by quantitative reverse transcription PCR (qRT-PCR), microarray, and next-generation sequencing (NGS) which requires time-consuming pre-treatment, manual operation, and a stand-alone instrument. To overcome these disadvantages, miRNA testing has been developed using the automated analyzers routinely used in clinical laboratories. An isothermal DNA amplification reaction was adapted to a fully automated immunoassay analyzer that conducts extraction, amplification, and detection processes at 37 °C in 44 min. In a reaction vessel, a pre-designed single-stranded signal DNA was amplified in the presence of miRNA, using DNA templates, DNA polymerase, and nicking endonuclease. Then, the amplified signal DNA was hybridized by one DNA probe attached to a magnetic particle and another DNA probe labeled with acridinium ester. After the chemiluminescence reaction, luminescence intensity was automatically measured. The automated assays of cancer-related miRNAs were implemented on the analyzer with throughput of 66 tests per hour. In the assays with one-step amplification, three miRNAs (miR-21-5p, miR-18a-5p, and miR-500a-3p) at concentrations lower than 100 fM were automatically detected and the cross reactivity for miR-21-5p with fifteen similar miRNAs was not higher than 0.02%. In the assay with two-step amplification, detection sensitivity and amplification rate for miR-21-5p were 3 fM and 103-fold, respectively. The coefficient of variations (CVs) in the measurement at the target concentrations from 5 fM to 1000 pM were less than 8%. Furthermore, we also achieved automated nucleic acid detection in human serum. The proposed fully automated miRNA assays showed high sensitivity, low cross reactivity, and reproducibility suitable for clinical use.

**Electronic supplementary material:**

The online version of this article (10.1007/s00216-019-01878-z) contains supplementary material, which is available to authorized users.

**Figure:**
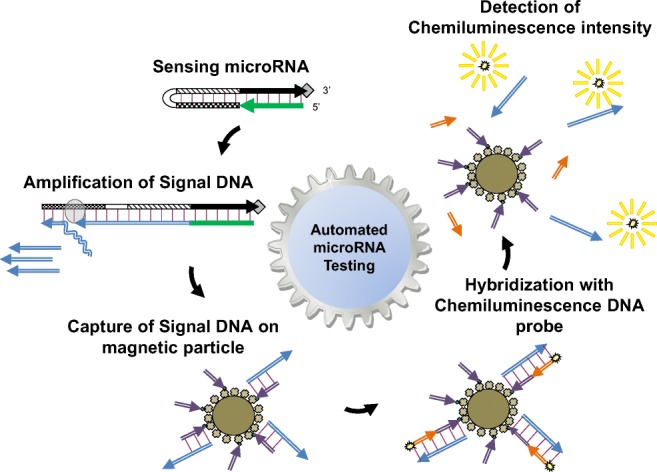
Graphical abstract

## Introduction

MicroRNAs (miRNAs) are functional small non-coding RNAs of 19–24 nucleotides that are involved in regulating many biological processes, including proliferation, differentiation, apoptosis, and development [1, 2]. The aberrant expression of miRNAs is closely related to cancer [3–6]. The miRNAs are stable in blood because most of miRNAs are bound to protective proteins such as Argonaute complexes and some miRNAs are inside or outside membrane vesicles such as exosomes [7–10]. Recently, circulating miRNAs have attracted attention as new potential biomarkers for cancer diagnosis, prognosis, and detection of recurrence [11–16]. However, the measurement of miRNAs is still challenging because miRNAs have cumbersome characteristics: short length (19–24 nucleotides), low concentration, and sequence homology among the miRNA family [17]. Research activities have been conducted to develop a miRNA quantification method for practical use [18–21].

The current major measurement methods of miRNAs are quantitative reverse transcription PCR (qRT-PCR), microarray, and next-generation sequencing (NGS) [2, 22]. While both microarray and NGS are suitable for screening and discovery purposes, qRT-PCR is still the first choice for validation and clinical tests with a high number of samples [16]. The qRT-PCR has been preferably used due to its high sensitivity and high specificity [2], but qRT-PCR in clinical laboratories has several disadvantages. First, a large qRT-PCR instrument used only for detecting nucleic acids is needed [23, 24]. Second, measurement cost is high due to the expensive instrument and the manual operation required for extraction and purification processes [25, 26]. Third, throughput is decreased by the long purification process due to blood coagulation under high-temperature conditions [26]. These disadvantages of qRT-PCR make it difficult to introduce miRNAs into clinical laboratories as cancer diagnostic markers [27, 28]. In addition, the manual operation has risk of human error.

Fully automated immunoassay analyzers are widely used in clinical laboratories to measure various proteins and chemical compounds as diagnostic markers in biological samples including serum, plasma, and urine [29–31]. In most fully automated immunoassay analyzers, luminescence intensity of chemiluminescent substrates is detected, such as acridinium ester, ruthenium complex, and luminol, after the labeled antibodies or antigens bind to the analytes [32]. The existing fully automated immunoassay analyzers can be used to process and measure miRNAs in serum under physiological temperature conditions. This avoids blood coagulation and the long purification process, eliminates the need to perform manual extraction processes, and eliminates the need to transfer the sample to the stand-alone qRT-PCR instrument. Implementation of miRNA testing on the automated immunoassay analyzers will lead to shorter test time and cost reduction and thus is significantly advantageous both for medical facilities and patients [27, 28].

According to the meta-analysis of clinical studies about miR-21-5p as a gastric cancer marker in serum and plasma, the cutoff concentrations of miR-21-5p were 37.3 fM, 50 fM, and 59.5 fM at three sites [13]. Therefore, a miR-21-5p assay needs a detection sensitivity around 50 fM. A previous study reported a detection sensitivity of miRNA at 1 pM using an acridinium-labeled antibody to a heterohybrid of miRNA and DNA on a fully automated immunoassay analyzer without DNA amplification [27]. To establish a more sensitive miRNA measurement, development of a method for DNA amplification is needed. However, the clinically approved methods of DNA amplification, such as loop-mediated isothermal amplification (LAMP) and strand displacement amplification (SDA), require the high reaction temperature at 60 °C and 54 °C, respectively [33, 34]. Although rolling circle amplification (RCA) achieves DNA amplification at 30–37 °C, it requires several hours for ligation reaction of padlock probe before DNA amplification [35, 36].

We recently proposed a novel isothermal DNA amplification method, *l*ow-*te*mperature *am*plification (L-TEAM), and demonstrated leak-free amplification [37]. In this method, pre-designed signal DNA is amplified exponentially in the presence of a target nucleic acid at constant 37 °C with the use of two DNA templates. In the present study, we adapted the miRNA measurement via L-TEAM for use with a fully automated immunoassay analyzer.

The exponential DNA amplification using immunoassay analyzers could lead to false positive results because the automated immunoassay analyzers are open systems. There is a possibility that the amplified signal sequence may contaminate other reaction vessels via a pipette or aerosol in the analyzer. In the reaction design of exponential amplification including PCR and L-TEAM, in which the end product as an amplified signal triggers amplification of the end product itself, contamination severely interferes amplification results. To minimize potential false positive results due to contamination, we modified L-TEAM to a cascade reaction that consists only of linear amplification. In the cascade reaction, successive sequence conversion of the generated single-stranded DNAs (ssDNAs) reduces the risk of false positive results by avoiding self-triggered exponential amplification and achieves higher amplification rates in comparison to one-step linear amplification.

In this paper, we first describe the automated assays for three miRNAs, i.e., miR-21-5p, miR-18a-5p, and miR-500a-3p, that were reported as cancer diagnostic markers [11, 12, 14], implemented on a fully automated immunoassay analyzer with one-step amplification reaction. Then, we report the assay performances upon the detection of miR-21-5p and miR-200 families that were reported as colorectal cancer markers [38, 39] on the analyzer with two-step amplification reaction.

## Materials and methods

### Materials for amplification and detection

All oligodeoxyribonucleotides and oligoribonucleotides were synthesized by Gene Design (Ibaraki, Japan). The Converter DNA and Cascade DNA were purified via ion exchange HPLC. The sequences of oligodeoxyribonucleotides and oligoribonucleotides and the chemical modification are described in Electronic Supplementary Material (ESM) Table S1. Bst DNA Polymerase, Large Fragment, Nb.BbvCI, NEB Buffer 2 (final concentration of 10 mM Tris-HCl, 50 mM NaCl, 10 mM MgCl_2_, 1 mM DTT, 0.1% Tween 20, pH 7.9), and dNTPs were purchased from New England Biolabs Japan (Tokyo, Japan). Streptavidin-coated magnetic particle, Dynabeads^®^ M-270 Streptavidin was purchased from Thermo Fisher Scientific K. K. (Kanagawa, Japan). The magnetic particles are uniform in size, having a diameter of 2.8 μm (https://www.thermofisher.com/jp/ja/home/brands/product-brand/dynal/streptavidin-coupled-dynabeads.html).

### Scheme of signal DNA amplification

**Fig. 1:**
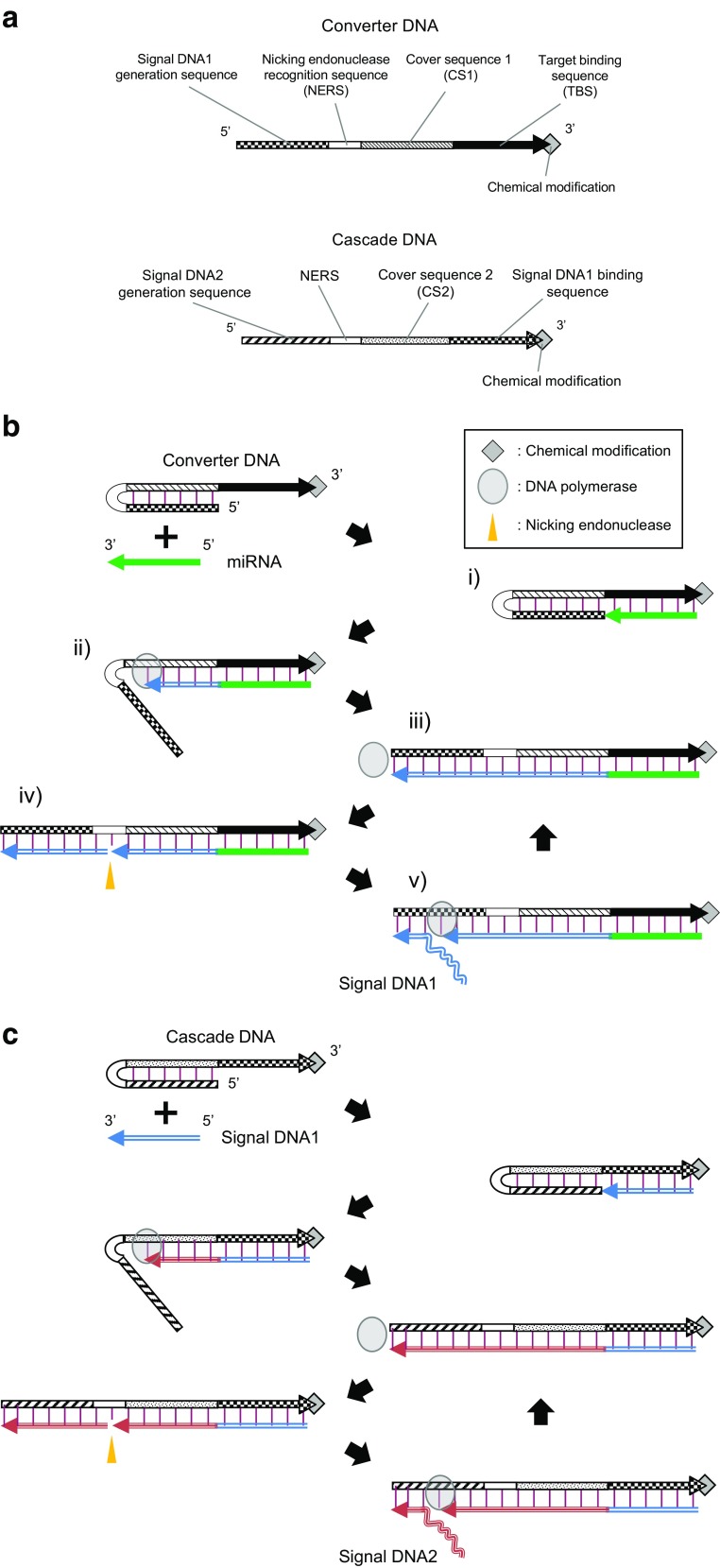
The schematic illustrations of **a** the sequence domains of Converter DNA and Cascade DNA, **b** linear signal DNA amplification reaction in the one-step amplification assay or the first step of the two-step amplification assay, and **c** linear signal DNA amplification reaction in the second step of the two-step amplification assay. Red vertical lines represent base pairings

The isothermal one-step signal DNA amplification (one-step amplification) reaction for miRNA detection requires one DNA template called Converter DNA, DNA polymerase having strand displacement activity, nicking endonuclease, dNTPs, and a target miRNA. Converter DNA comprises Signal DNA1 generation sequence, nicking endonuclease recognition sequence (NERS), cover sequence 1 (CS1) which is complementary to Signal DNA1 generation sequence, and target binding sequence (TBS) which is complementary to a target miRNA in the 5′ to 3′ direction (Fig. 1a). At the reaction temperature, Converter DNA forms a hairpin structure comprising an 18-bp stem and avoids binding of Signal DNA1 released from a Converter DNA to the Signal DNA1 generation sequence of another Converter DNA.

When miRNA hybridizes with the TBS of Converter DNA, DNA extension occurs from the 3′ terminal of miRNA by DNA polymerase (steps i and ii in Fig. 1b). During DNA extension, the hairpin structure is opened due to the strand displacement activity of DNA Polymerase, and the DNA strand complementary to Converter DNA is generated (step iii). Then, nicking endonuclease recognizes its recognition sequence in the double-stranded form. Upon nicking reaction, Signal DNA1 is generated (step iv). After that, DNA extension occurs again from the 3′ terminal of nicked position. As a consequence, the formerly generated Signal DNA1 is released because of the strand displacement activity of DNA Polymerase (step v). These polymerization and nicking reactions repetitively occur and linearly amplify Signal DNA1, only in the presence of miRNA without the needs of reverse transcription reaction and tag-adding reaction for generating primer-binding sequence that are required in qRT-PCR.

The isothermal two-step cascade signal DNA amplification (two-step amplification) reaction is also achieved by layering two consecutive linear signal DNA amplification reactions (Fig. 1b, c). In the second step reaction, hybridization of Signal DNA1 with the Signal DNA1 binding sequence of Cascade DNA further triggers the next linear amplification of Signal DNA2, whose sequence is different from that of Signal DNA1, via the repetitive polymerization and nicking reaction similarly to the first step reaction.

### Preparation of Capture DNA probe attached to magnetic particles

Capture DNA probe (13 nucleotides (nt) in length) labeled with biotin-triethylene glycol spacer at its 3′ end was incubated with streptavidin-coated magnetic particles for 30 min at a room temperature. After washing the magnetic particles under magnetic attraction, a 0.05% (*w*/*v*) particle solution was prepared.

### Preparation of Chemiluminescence DNA probe

Chemiluminescence DNA probe (10 nt) modified with amino group-six-carbon spacer at its 5′ end was reacted with NHS-conjugated acridinium ester provided by Abbott Laboratories (IL, USA) for overnight at room temperature. The resulting Chemiluminescence DNA probe labeled with acridinium ester was separated by reversed-phase HPLC (Nihon Waters K.K., Tokyo, Japan) and diluted to 100 nM.

### Automated miRNA detection on the analyzer

**Fig. 2:**
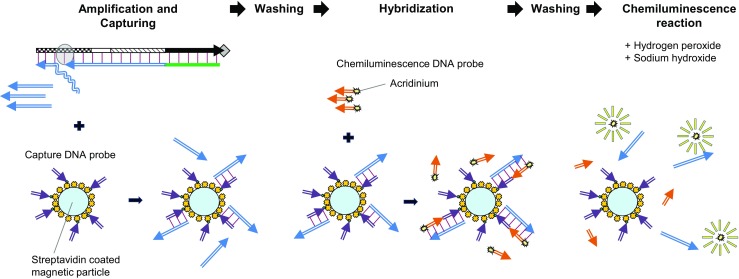
The schematic illustrations of capturing of signal DNA followed by chemiluminescence reaction on the immunoassay analyzer

A fully automated immunoassay analyzer, ARCHITECT i system (Abbott Japan, Tokyo, Japan) is used for measuring proteins and chemical compounds in serum, plasma, and urine as diagnostic markers in about 30 min at clinical laboratories [29]. In the present study, automated miRNA assays were implemented on the immunoassay analyzer as follows. One sample solution and six operational solutions were placed on the analyzer, and the miRNA assay was started. Initially, a 40-μL sample solution containing synthetic miRNA in 10 mM Tris-HCl and 0.01% BSA (pH 8.0) was dispensed into a reaction vessel. Then, a 50-μL solution containing detergent was mixed for 7 min at 37 °C on the fully automated analyzer. The role of detergent is to liberate miRNAs when the sample is serum. A 30-μL solution of this mixture was transferred to another reaction vessel. Next, a 40-μL solution of Converter DNA, a 40-μL pre-mixed solution of Bst DNA Polymerase, Large Fragment, and Nb.BbvCl, and a 20-μL dNTP solution were dispensed into the reaction vessel and incubated for 7 min at 37 °C. In this amplification reaction mixture containing Bst DNA Polymerase, Large Fragment, and Nb.BbvCI at 0.07 and 0.09 units/μL, respectively, hybridization of target miRNA to Converter DNA primes DNA extension and Signal DNA1 is linearly amplified by the repetitive polymerization and nicking reaction. After 7 min, 70-μL of this reaction mixture was transferred to the other reaction vessel. A 50-μL solution of Capture DNA probe attached to the magnetic particle was dispensed in the reaction vessel and incubated for 18 min at 37 °C. In this amplification and capturing reaction mixture containing Bst DNA Polymerase, Large Fragment, and Nb.BbvCI at 0.04 and 0.05 units/μL, respectively, amplification of Signal DNA1 continues and hybridization of Signal DNA1 to a Capture DNA probe concurrently occurs (Fig. 2). After washing the magnetic particles under magnetic attraction, a 50-μL solution of acridinium-labeled Chemiluminescence DNA probe was dispensed and incubated for 4 min at 37 °C. In this hybridization reaction mixture, Chemiluminescence DNA probe hybridizes to Signal DNA1 captured by Capture DNA probe on the magnetic particle. After washing the magnetic particles again under magnetic attraction, pre-trigger solution containing hydrogen peroxide and trigger solution containing sodium hydroxide of ARCHITECT i system (Abbott Japan) were dispensed and incubated for 18 s at 37 °C. The chemiluminescence of acridinium at the wavelengths in the range of 400 to 500 nm was measured by the optical system of the analyzer. In principle, the intensity of chemiluminescence is proportional to the amount of amplified Signal DNA1. In the one-step amplification assays, Converter DNA-21, Converter DNA-18, and Converter DNA-500, which were designed for the corresponding target miRNAs (miR-21-5p, miR-18a-5p, and miR-500a-3p), were used. The final concentrations of Converter DNA and each dNTP were 1.4 nM and 100 μM, respectively. In the two-step amplification assays for miR-21-5p and miR-200 family, the reaction was performed similarly as the above described with the Converter DNAs, each having the TBS for the corresponding target miRNA (miR-21-5p, miR-200a, miR-200b, and miR-200c), except for the final concentrations of Cascade DNA and each dNTP were 4.2 nM and 10 μM, respectively.

### Automated nucleic acid detection in human serum on the analyzer

The ssDNA having the sequence the same as miR-21-5p, termed miD-21-5p, was spiked directly into normal human serum. The sample was measured via the two-step amplification assay for miR-21-5p.

## Results and discussion

### Automated miRNA detection with one-step amplification on the analyzer

**Fig. 3:**
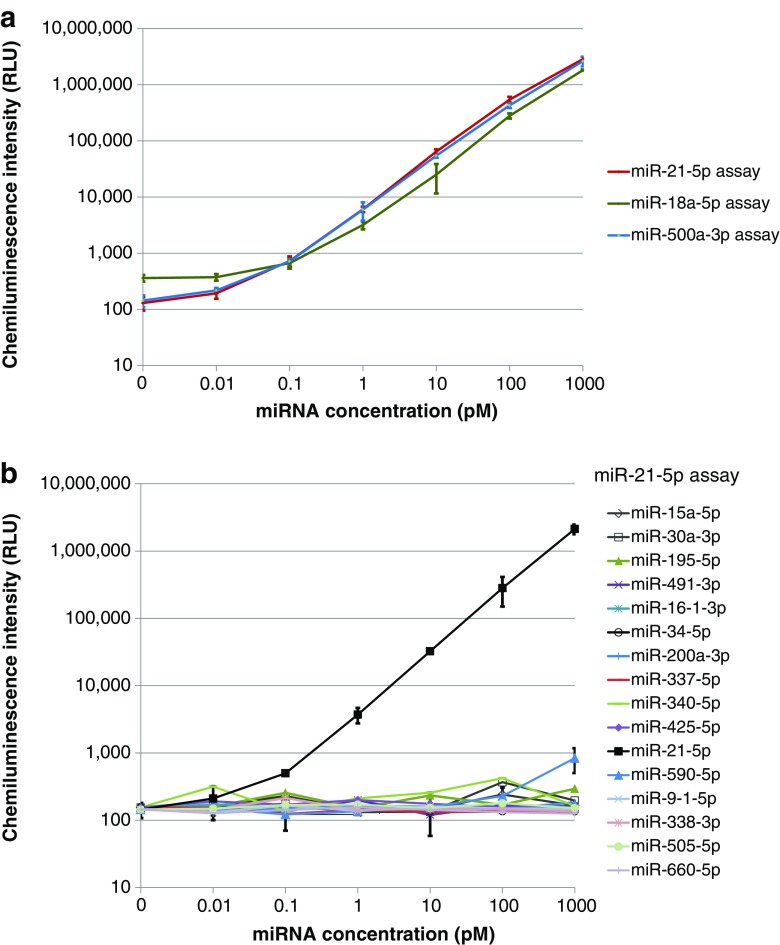
**a** Dose responses in the respective one-step amplification assays for miR-21-5p, miR-18a-5p, and miR-500a-3p on the analyzer. The bar shows ± 2 standard deviations. Each sample was measured in triplicate except for the blank sample (replicate (rep.) = 5). **b** Cross reactivity test of fifteen human miRNAs which have sequences similar to that of miR-21-5p in the one-step amplification assay for miR-21-5p on the analyzer. The bar shows ± 2 standard deviations. Each miRNA sample was measured in triplicate except for the blank sample (rep. = 5)

We measured different concentrations of synthetic miR-21-5p from 0.01 to 1000 pM in triplicate, and 0 pM in replicates of 5 via the one-step amplification assay for miR-21-5p using Converter DNA-21. The Converter DNA was designed to form a hairpin structure by the introduction of cover sequence as shown in Fig. 1. The hairpin structure accelerates DNA amplification by preventing signal DNA released into the solution from hybridizing to signal generation sequence of intact Converter DNA (see ESM Methods, ESM Results, ESM Fig. S1, Table S2). The assay results were obtained in 44 min after initial sample dispense with throughput of 66 tests per hour. The resulting dose-response curve of the miR-21-5p assay is shown in Fig. 3a. The coefficient of variations (CVs) of chemiluminescence intensity (CI) for the target at concentrations of 1 pM and higher were less than 6%. The CVs of CI even at low target concentrations both of 10 and 100 fM were 10%, and that of the background CI at 0 pM was 13%. The detection sensitivity was in the range from 10 to 100 fM. Similarly, we also measured miR-18a-5p and miR-500a-3p on the analyzer with Converter DNA-18 and Converter DNA-500, respectively (Fig. 3a). For clarity, each dose-response curve was separately shown in ESM Fig. S2A–C.

The one-step amplification assays for miR-18a-5p and miR-500a-3p, which were developed by changing the TBS of Converter DNA-21, exhibited comparable sensitivity as shown in the dose-response curves (Fig. 3a). In the present assay system, the target miRNA is easily altered only by changing the TBS of Converter DNA since miRNA is converted to the common signal DNA to be detected with the common Capture DNA probe and Chemiluminescence DNA probe. More than a hundred circulating miRNAs in blood have been so far identified as diagnostic, prognostic, or predictive biomarkers for different types of cancers [15]. The proposed assay allows the use of a common detection system and promotes the development of multiple miRNA assays.

Next, we investigated the cross reactivity of the one-step amplification assay for miR-21-5p to evaluate the sequence specificity. We selected fifteen human miRNAs which have sequences similar to that of miR-21-5p according to the miRNA database, miRBase [2, 40]. The numbers of bases identical to those in miR-21-5p sequence ranged from 8 to 14 (ESM Table S3). We measured the samples of each synthetic miRNA at the concentrations from 0 to 1000 pM. Concentrations of synthetic miRNAs which interfered with the assay of miR-21-5p were estimated by the dose-response curve of miR-21-5p. The concentration was defined as interfering miR-21-5p concentration (Fig. 3b). The cross reactivity was evaluated with the ratio of the interfering miR-21-5p concentration to each synthetic miRNA concentration. No cross reactivity was observed for fourteen miRNAs (miR-15a-5p, miR-30a-3p, miR-195-5p, miR-491-3p, miR-16-1-3p, miR-34a-5p, miR-200a-3p, miR-337-5p, miR-340-5p, miR-425-5p, miR-9-1-5p, miR-338-3p, miR-505-5p, miR-660-5p) (Fig. 3b). At the highest, 0.02% of cross reactivity was found in miR-590-5p at the concentration of 1000 pM, which has 13 bases identical to those in miR-21-5p sequence. These results indicated high sequence specificity of the proposed miR-21-5p assay.

### Automated miR-21-5p detection with two-step amplification on the analyzer

**Fig. 4:**
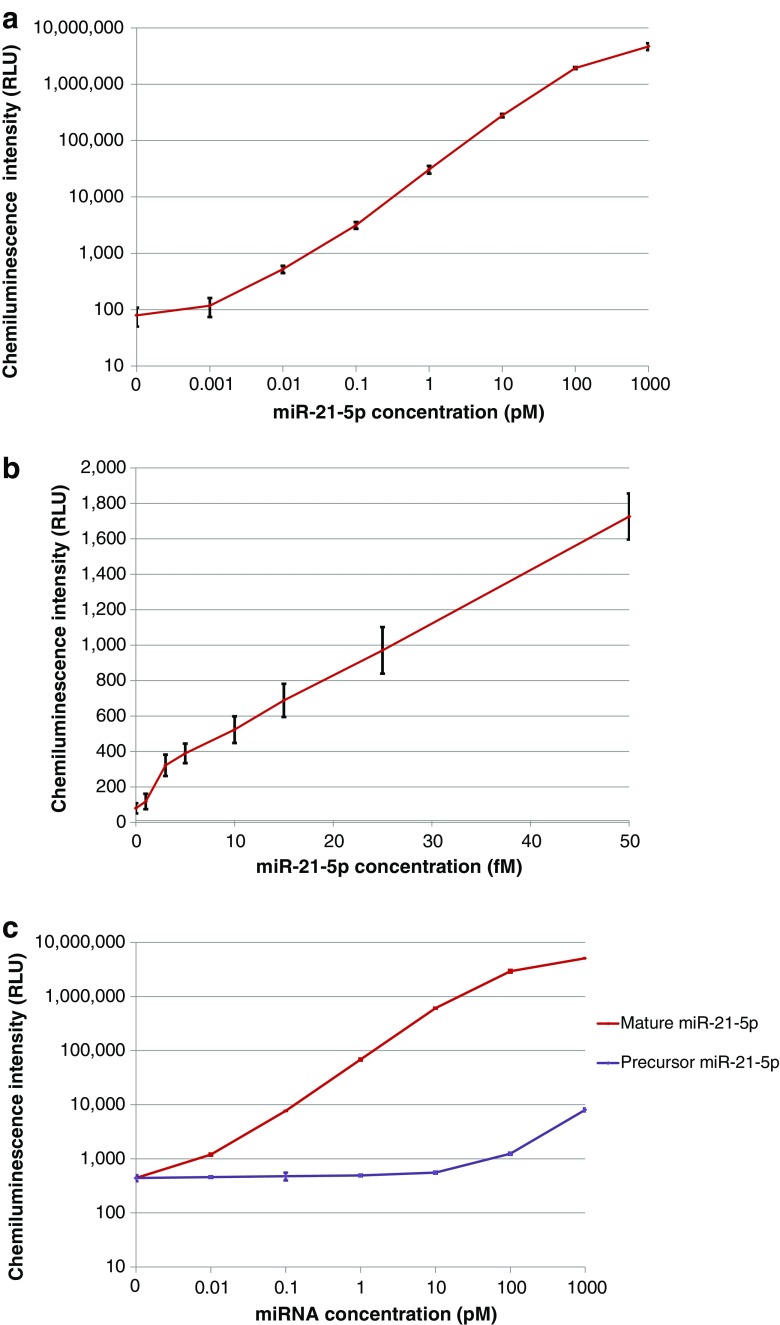
**a** Dose response in the two-step amplification assay for miR-21-5p at the concentrations from 0 to 1000 pM on the analyzer. The bar shows ± 2 standard deviations. Each sample was measured in replicates of 5. **b** Dose response for the detection sensitivity test in the two-step amplification assay for miR-21-5p at the concentrations from 0 to 50 pM on the analyzer. The bar shows ± 2 standard deviations. Each sample was measured in replicates of 20. **c** Cross reactivity test of precursor miR-21-5p in the two-step amplification assay for miR-21-5p on the analyzer. The bar shows ± 2 standard deviations. Each sample was measured in triplicate except for the blank sample (rep. = 5)

The detection sensitivity of the one-step amplification assay for miR-21-5p in the range from 10 to 100 fM was not sufficient because the cutoff concentration of miR-21-5p in serum and plasma for cancer diagnosis is around 50 fM [13]. We extended the one-step amplification assay to the two-step amplification assay by layering linear amplification reactions [41]. We measured different concentrations of synthetic miR-21-5p from 0 to 1000 pM in replicates of 5 on the analyzer. The obtained dose-response curve is shown in Fig. 4a. The curve fitting of the dose-response curve with fitted equation and correlation coefficient is shown in ESM Fig. S3A. For determining the precise detection limit, we further measured different concentrations of synthetic miR-21-5p from 0 to 50 fM in the samples in replicates of 20 (Fig. 4b). The sample with 0-fM (blank) and 3-fM miR-21-5p showed the mean CI of 79 relative light unit (RLU) (standard deviation (SD) 15) and 322 RLU (SD 30), respectively. The 3 fM of miR-21-5p was distinguished from the blank sample (*P* (two-tailed unpaired *t* test) < 10^−21^). The two-step amplification assay achieved the detection sensitivity value of 3 fM for miR-21-5p, significantly lower than the cutoff concentrations in serum around 50 fM [13]. This highly sensitive detection is attributed not only to the introduction of cover sequence and layering linear amplification reactions, but also to the application of chemiluminescence reaction with Capture DNA probe and Chemiluminescence DNA probe emulating the common sandwich assay in immunoassay. This reaction design is suitable for the fully automated immunoassay analyzer.

In Fig. 4a, the CVs of CI for the target at concentrations from 0.01 to 1000 pM were less than 8%. The CV of CI even at low target concentration of 0.001 pM and that of the background CI at 0 pM were both 18%. In Fig. 4b, the CVs of CI for the target at concentrations of 3 fM were 9%, and those at five concentrations from 5 to 50 fM were less than 8%. These results indicated the high sensitivity and reproducibility of the assay.

Amplification rates as the ratios of amplified Signal DNA2 concentration to the target miR-21-5p concentration were estimated by using the calibration curve of Signal DNA2. The calibration curve of Signal DNA2 was generated in the measurement with serially diluted Signal DNA2 in 10 mM Tris-HCl, 0.01% BSA (pH 8.0) (ESM Fig. S3B, C). The mean amplification rate for the target concentrations from 0.1 to 10 pM was 103-fold. For comparison, we also estimated the amplification rate in the one-step amplification assay (ESM Fig. S4), based on the results shown in Fig. 3a. The obtained mean amplification rate for the target miR-21-5p at concentrations from 0.1 to 10 pM was 26-fold. The cascading of signal DNA generation achieved the higher amplification rate in the two-step amplification assay for miR-21-5p than that in the one-step amplification assay.

We also investigated whether precursor miR-21-5p (60 nt), which contains mature miR-21-5p sequence and forms a secondary structure, and mature miR-21-5p (22 nt) were distinguished in the two-step amplification assay. The samples of synthetic precursor miR-21-5p and mature miR-21-5p at the concentrations from 0 to 1000 pM were measured. The cross reactivity evaluated as the ratio of interfering mature miR-21-5p concentration to precursor miR-21-5p concentration was, at the highest, 0.01% at the concentration of 1000 pM (Fig. 4c). The result indicated that the miR-21-5p assay with Converter DNA-21 preferably detected mature miR-21-5p than precursor miR-21-5p.

### Automated miR-200 family detection with two-step amplification on the analyzer

**Fig. 5:**
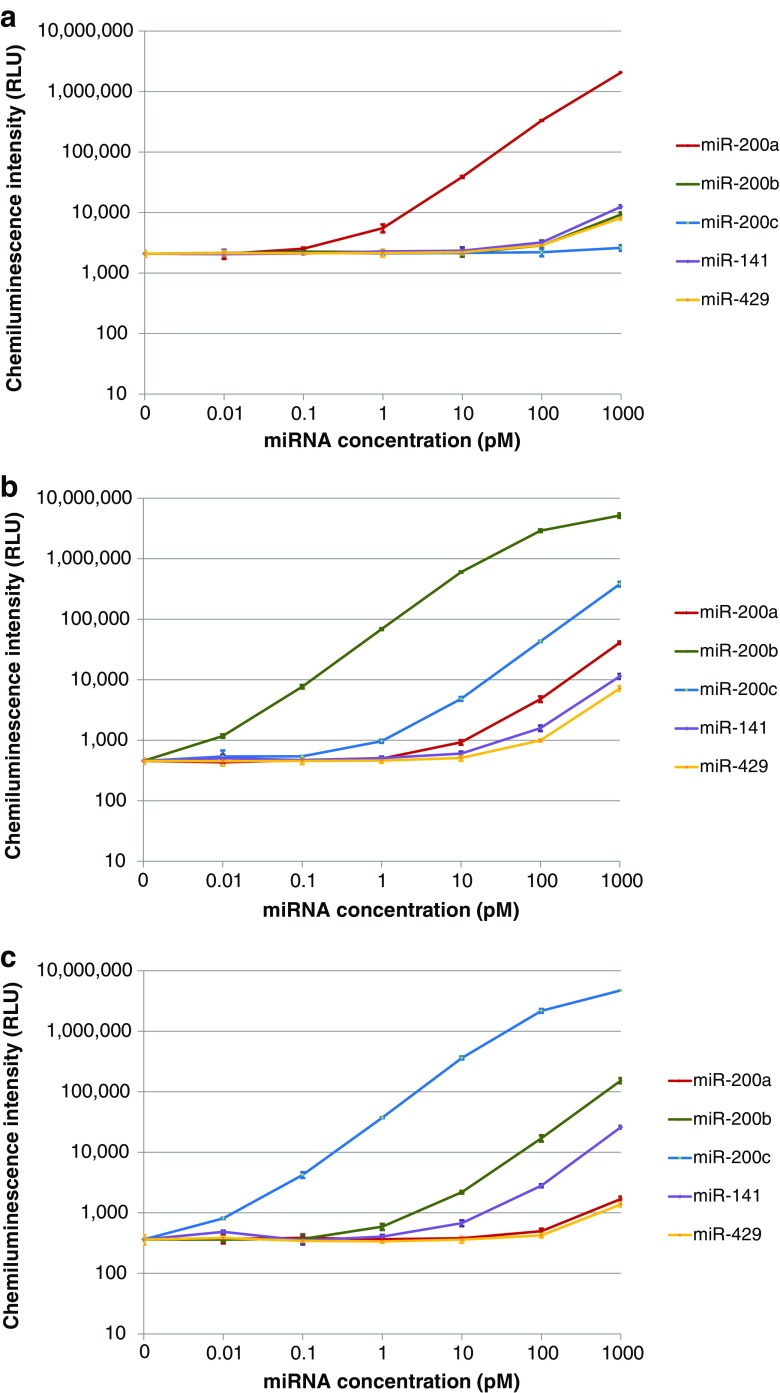
**a** Cross reactivity test of four miRNAs (miR-200b, miR-200c, miR-141, miR-429) in miR-200 family in the two-step amplification assay for miR-200a on the analyzer. The bar shows ± 2 standard deviations. Each sample was measured in triplicate except for the blank sample (rep. = 5). **b** Cross reactivity test of four miRNAs (miR-200a, miR-200c, miR-141, miR-429) in the two-step amplification assay for miR-200b on the analyzer. The bar shows ± 2 standard deviations. Each sample was measured in triplicate except for the blank sample (rep. = 5). **c** Cross reactivity test of four miRNAs (miR-200a, miR-200b, miR-141, miR-429) in the two-step amplification assay for miR-200c on the analyzer. The bar shows ± 2 standard deviations. Each sample was measured in triplicate except for the blank sample (rep. = 5)

The miR-200 family consists of miR-200a, miR-200b, miR-200c, miR-141, and miR-429 which have highly homologous sequences (ESM Table S1B) [39]. We investigated the cross reactivity of the two-step amplification assays for miR-200a, miR-200b, and miR-200c by evaluating the ratio of interfering miRNA concentrations of each assay to each synthetic miRNA concentration. The samples of synthetic miRNAs, each containing miR-200a, miR-200b, miR-200c, miR-141, or miR-429 at the concentrations from 0 to 1000 pM, were measured in three respective assays with Converter DNAs having TBS for miR-200a, miR-200b, and miR-200c. In the assay for miR-200a, the cross reactivity values were not higher than 0.20, 0.01, 0.30, and 0.19% for miR-200b, miR-200c, miR-141, and miR-429, respectively (Fig. 5a). In the assay for miR-200b, the cross reactivity values were not higher than 0.06, 0.64, 0.02, and 0.01% for miR-200a, miR-200c, miR-141, and miR-429, respectively (Fig. 5b). In the assay for miR-200c, the cross reactivity values were 0.003, 0.44, 0.07, and 0.002% for miR-200a, miR-200b, miR-141, and miR-429, respectively (Fig. 5c). The similar miRNA sequences did not cause the false positive signal higher than 0.64%, showing high sequence specificity of the two-step amplification assays for miR-200 family detection.

These results indicated that our miRNA assays with two-step amplification on the analyzer have high reproducibility and sequence specificity and could be clinically useful in cancer detection.

### Automated nucleic acid detection in human serum on the analyzer

**Fig. 6:**
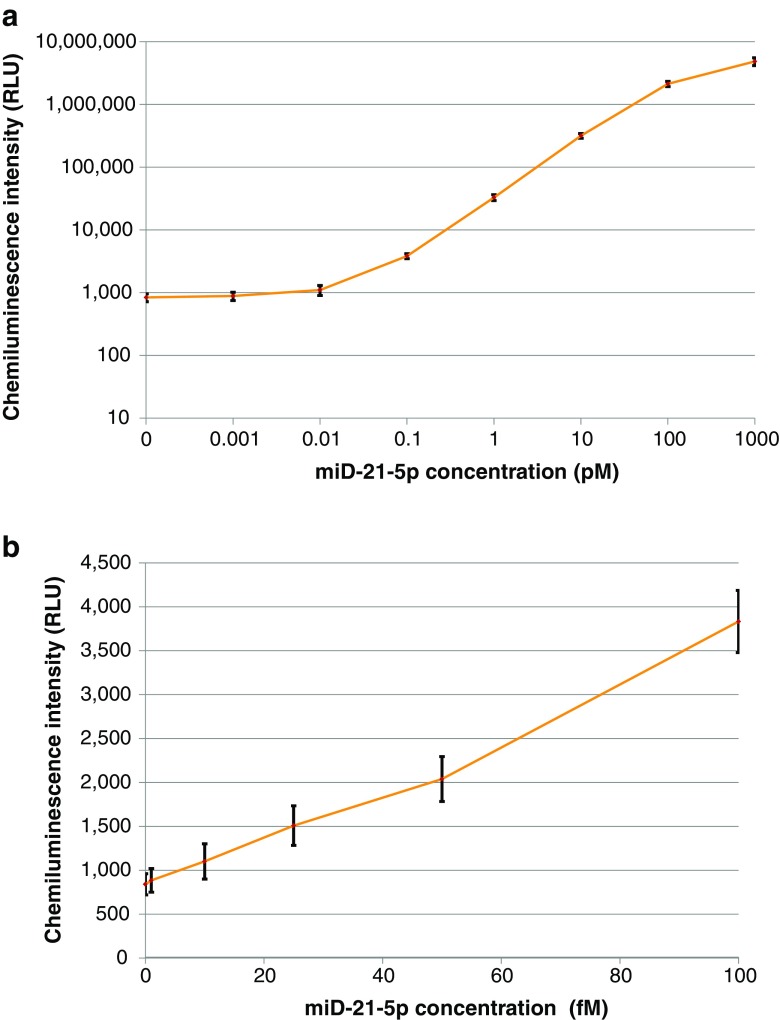
**a** Dose response on the spiked miD-21-5p, which was the single-stranded DNA with the same sequence as miR-21-5p, in human serum at the concentrations from 0 to 1000 pM in the two-step amplification assay for miR-21-5p on the analyzer. The bar shows ± 2 standard deviations. Each sample was measured in replicates of 10 except for the blank serum sample (rep. = 20). **b** Dose response for the detection sensitivity test on the spiked miD-21-5p in human serum at the concentrations from 0 to 100 fM in the two-step amplification assay for miR-21-5p on the analyzer. The bar shows ± 2 standard deviations. Each sample was measured in replicates of 10 except for the blank serum sample (rep. = 20)

We performed the two-step amplification assay in normal human serum containing the ssDNA on the analyzer. MiD-21-5p spiked in serum samples at different concentrations from 0.001 to 1000 pM and a blank at 0 pM were measured in replicates of 10 and 20, respectively, on the automated analyzer. The obtained dose-response curve is shown in Fig. 6a. The curve fitting of the dose-response curve with fitted equation and correlation coefficient is shown in ESM Fig. S5A.

To determine the sensitivity of the assay, we further measured different concentrations of miD-21-5p in serum samples from 1 to 100 fM in replicates of 10 and a blank at 0 fM in replicates of 20 (Fig. 6b). The mean CI calculated for the target miD-21-5p at the concentrations of 0 and 10 fM were 840 RLU (SD 60) and 1101 RLU (SD 100), respectively. The 10 fM of miD-21-5p in serum was distinguished from the blank serum sample (*P* (two-tailed unpaired *t* test) < 10^−8^). The CVs of CI in the measurement with the target miD-21-5p in serum at concentrations from 0 to 1000 pM were less than 8% except for 9% at 0.01 pM, which again represented the high reproducibility of the assay. The mean amplification rate for the target concentrations from 0.1 to 10 pM was 126-fold (see ESM Fig. S5B, C). These results indicated that the two-step amplification assay for ssDNA in normal human serum had high detection sensitivity and reproducibility.

The physiological temperature condition of constant 37 °C eliminated the need to purify the miRNA due to blood clotting caused by high-temperature conditions. Also, detergent addition, which is required for detaching miRNAs from the protein or breaking down exosomes in human serum [42], was implemented in the fully automated process.

## Conclusions

The fully automated miRNA measurement was developed by adapting an isothermal DNA amplification reaction to the existing automated immunoassay analyzer, ARCHITECT i system. The reaction temperature at constant 37 °C eliminates the purification process and results in a testing time of 44 min and a throughput of 66 tests per hour. The proposed assay for a cancer-related miRNA showed high detection sensitivity, high reproducibility, and low cross reactivity. Implementation of the measurement of miRNAs as potential cancer markers together with immunoassays for the conventional protein markers on the single automated analyzer would allow the precise diagnosis and reduce the cost of nucleic acid test. We believe our work will enable early cancer diagnosis based on high throughput measurement of multiple biomarkers in full automation, which is useful both for patients and clinical laboratories in terms of testing time, cost, and accurate diagnosis.

## Electronic supplementary material

ESM 1 (DOCX 515 kb)
