# Supplementary material for: Measurement of microRNA with isothermal DNA amplification on fully automated immunoassay analyzers
Source: Anal Bioanal Chem. 2019 Jun 3;411(17):3789–800. doi: 10.1007/s00216-019-01878-z (PMC6595071; doi:10.1007/s00216-019-01878-z)
Supplement: Supplementary file 1 — (DOCX 515 kb) [file 216_2019_1878_MOESM1_ESM.docx]

**Analytical and Bioanalytical Chemistry**

**Electronic Supplementary Material**

**Measurement of microRNA with isothermal DNA amplification on fully automated immunoassay analyzers**

Makoto Komori, Ken Komiya, Takuma Shirakawa, Takamitsu J. Morikawa, Toru Yoshimura

**Sequences**

Oligodeoxyribonucleotide and oligoribonucleotide sequences used in the miRNA assays on the analyzer in the main text are shown in Table S1. Oligodeoxyribonucleotide sequences used in the supplementary experiment for investigating the effect of cover sequence introduction and comparison of nicking endonucleases are shown in Table S2. Sequences of miR-21-5p and its similarity to the fifteen human miRNAs used in the cross reactivity test are shown in Table S3.

**Methods**

**Investigation on the effect of cover sequence introduction to Converter DNA and comparison of nicking endonucleases**

We investigated the amplification behavior in the one-step amplification assay for miR-24-3p using Converter DNAs with and without cover sequence (Table S2). Amplification of Signal DNA was monitored by detecting the fluorescence emitted upon hybridization with the molecular beacon on a real-time PCR system, CFX 96 (BioRad Japan, Tokyo, Japan). The amplification reaction was performed at constant 37.0^o^C in 25-μL reaction mixtures of NEB Buffer 2 (final concentration of 10 mM Tris-HCl, 50 mM NaCl, 10 mM MgCl_2_, 1 mM DTT, 0.1% Tween 20, pH 7.9), with a target single-stranded DNA having the sequence same as miR-24-3p termed miD-24-3p, Converter DNAs with or without cover sequence, Bst DNA Polymerase, Large Fragment, Nt.AlwI or Nb.BbvCI, and dNTPs. Fluorescence detection was implemented at intervals of about 69 seconds for 120 times. The final concentrations of Bst DNA Polymerase, Large Fragment, Nt.AlwI or Nb.BbvCI, and dNTPs were 0.08 units/μL,0.1 units/μL, and 200 μM each, respectively. The final concentrations of the target DNA, Converter DNAs, and the molecular beacon were 1, 100, and 100 nM, respectively.

**Generation of the calibration curve for estimating the amplification rate**

The serially diluted Signal DNA solutions were spiked in 10 mM Tris-HCl, 0.01% BSA (pH 8.0) and automatically measured similarly to the corresponding miRNA assays with Converter DNA, DNA polymerase, nicking endonuclease, dNTPs, Capture DNA probe, and Chemiluminescence DNA probe as described in the main text.

**Results**

**The effect of cover sequence introduction to Converter DNA and comparison of nicking endonucleases in efficiency of amplification reaction**

Fluorescence intensity representing the Signal DNA amplification in the isothermal one-step amplification assay of the target miD-24-3p increased in the order of Converter DNA-24-1 without the cover sequence and with the recognition sequence of Nt.AlwI, Converter DNA-24-2 with the cover sequence and the recognition sequence of Nt.AlwI, and Converter DNA-24-3 with the cover sequence and the recognition sequence of Nb.BbvCI. (Fig. S1). We concluded that the introduction of the cover sequence to Converter DNA promoted the Signal DNA amplification and the amplification efficiency with Nb.BbvCI is higher than that with Nt.AlwI in the reaction at constant 37 ^o^C.

**Table S1** Oligodeoxyribonucleotide and oligoribonucleotide sequences used in the (**A**) one-step and (**B**) two-step amplification assays for miRNA measurement on the analyzer. The 3' terminals of Converter DNA and Cascade DNA were chemically modified with two consecutive inverted deoxythymidines, represented by (I)-(I), to avoid unexpected extension reactions. Since the GC-content of Chemiluminescence DNA probe 1 was low, Locked Nucleic Acid, which is an artificial nucleic acid having a bridged structure in the ribose portion, was introduced into it as indicated by the superscript L symbol for assuring high detection sensitivity. (B-TEG) and (NH_2_-C6) represent biotin with triethylene glycol spacer and primary amine with six carbon spacer, respectively. The underlined letters represent the recognition sequences of Nb.BbvCl

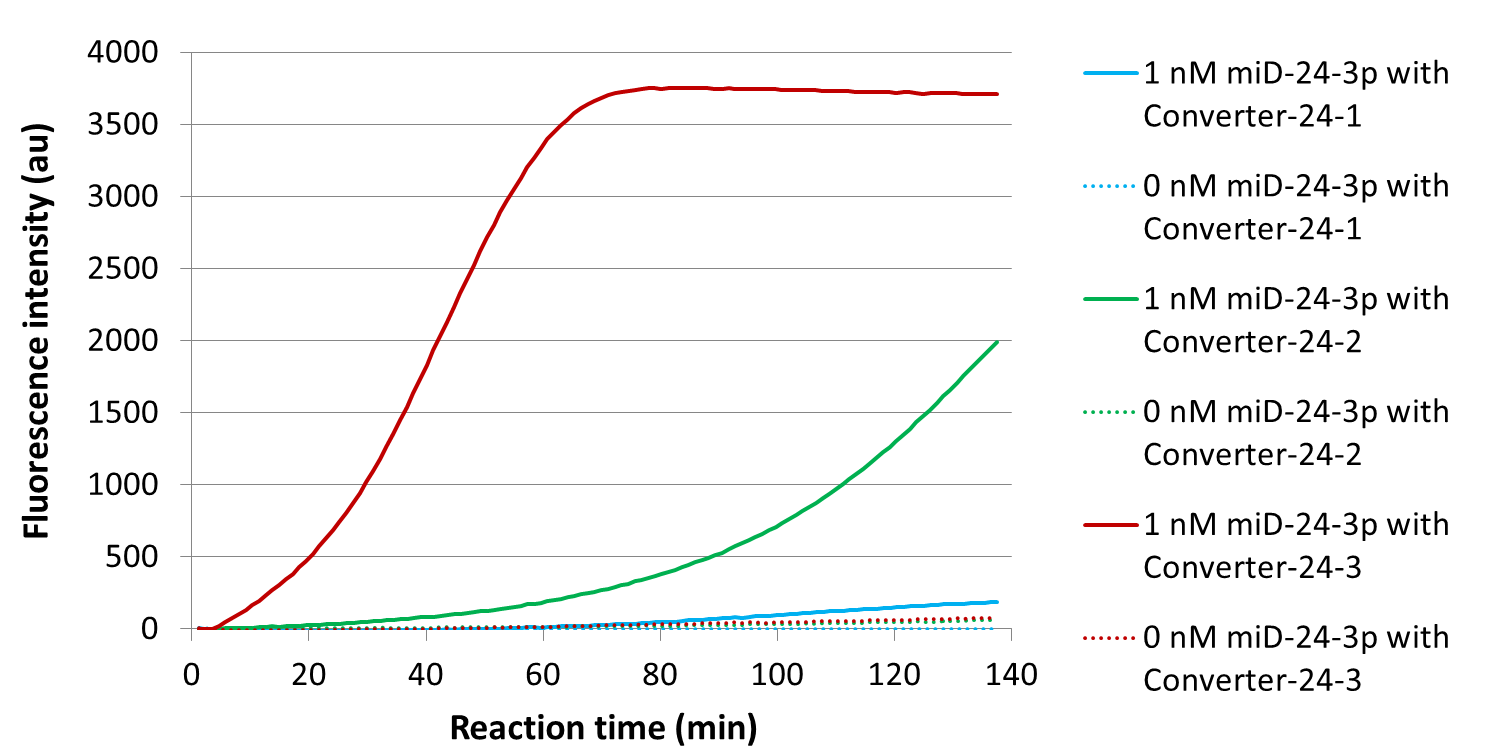


**Fig. S1** Mean fluorescence curves in the isothermal one-step amplification assay of the target miD-24-3p with Converter DNA-24-1 without the cover sequence and with the recognition sequence of Nt.AlwI, Converter DNA-24-2 with the cover sequence and the recognition sequence of Nt.AlwI, and Converter DNA-24-3 with the cover sequence and the recognition sequence of Nb.BbvCl. Each sample was measured in triplicate and averaged

**Table S2** Oligodeoxyribonucleotide sequences used in the supplementary experiment for investigating the effect of cover sequence introduction to Converter DNA and comparison of nicking endonucleases. The underlined and double-underlined letters of Converter-24-1, 24-2 and Converter-24-3 represent the recognition sequences of Nt.AlwI and Nb.BbvCl, respectively. Converter-24-1 has no cover sequence. Converter-24-2 and Converter-24-3 were designed to form the hairpin structure with 18-bp stems. (F) and (D) represent 6-carboxyfluorescein (6-FAM), and dabcyl, respectively. The molecular beacon, which was designed to form the hairpin structure with a 5-bp stem, has the sequence complementary to that of Signal DNA. Upon hybridization to the Signal DNA, the fluorophore, 6-FAM and the quencher, dabcyl attached to the 5′ and 3′ terminal ends of the molecular beacon becomes apart, resulting in fluorescence emission

**A**


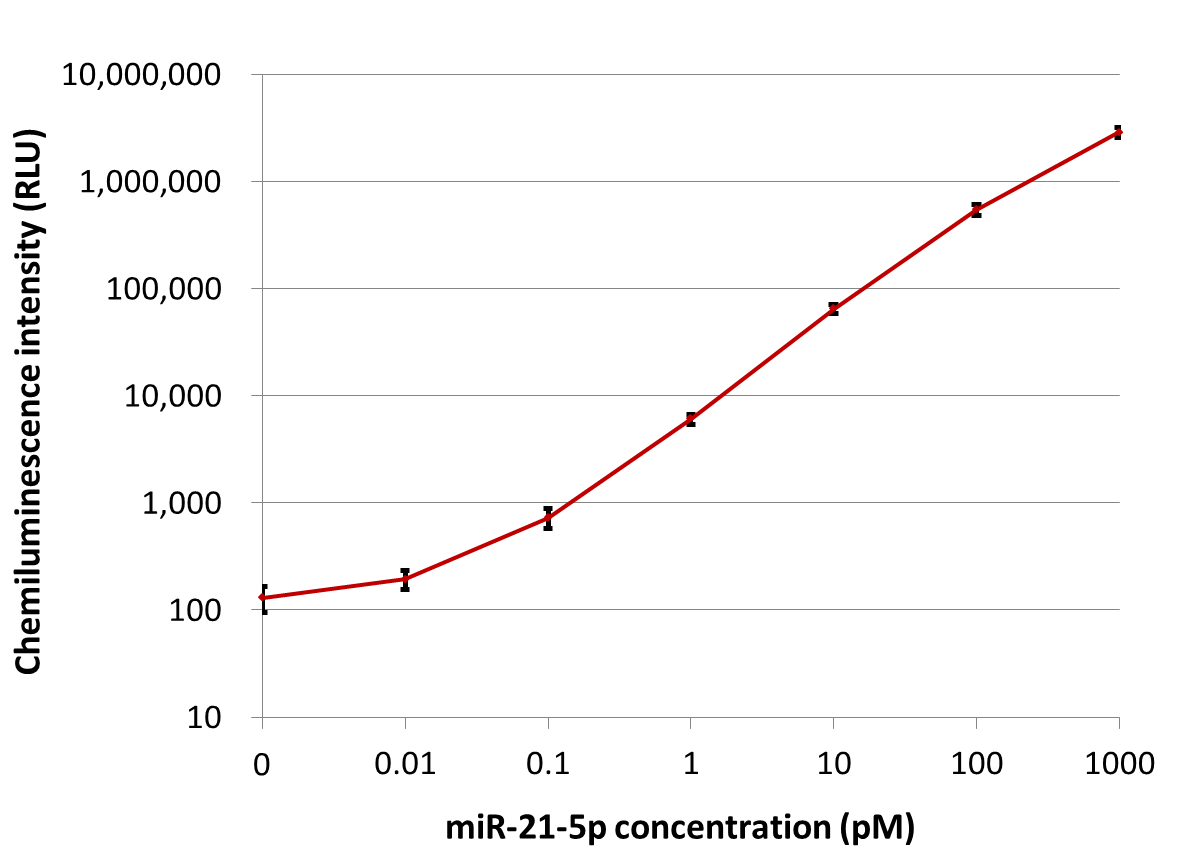


**B**


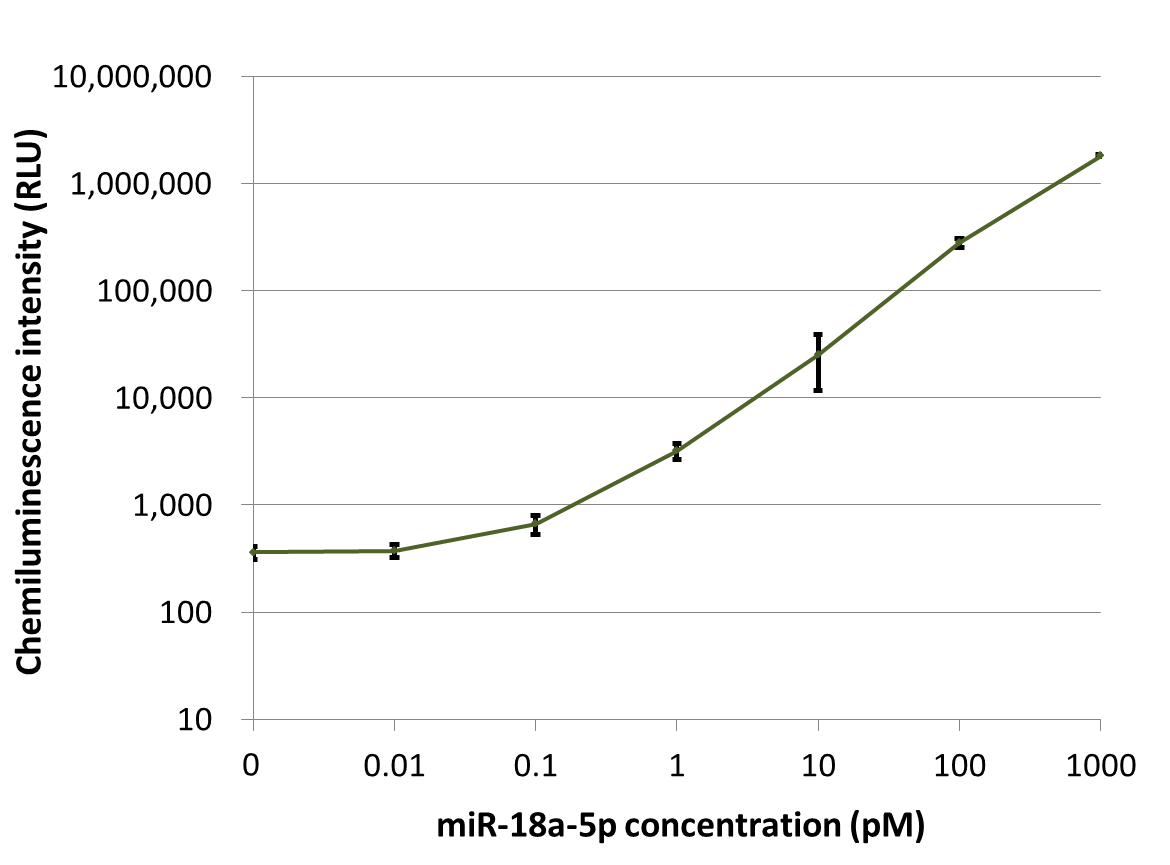


**C**


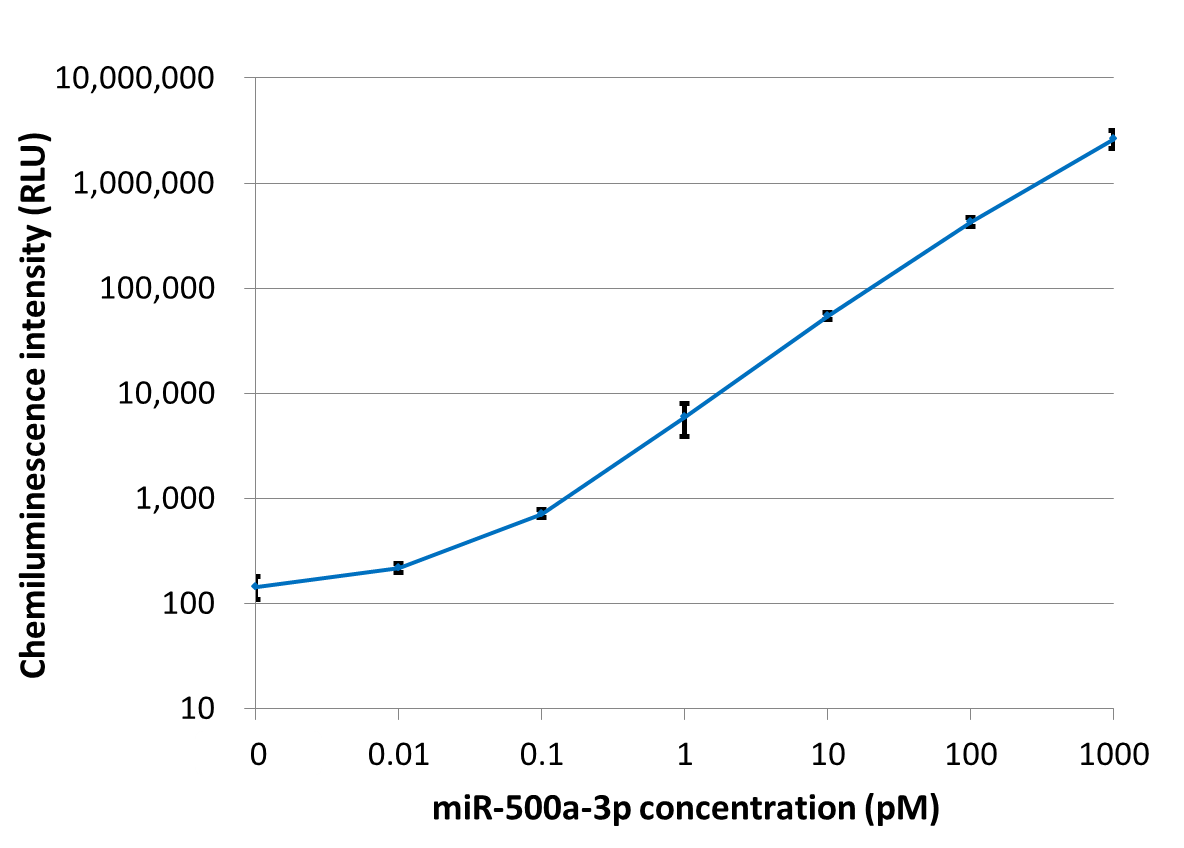


**Fig. S2** Separate dose response curves in the one-step amplification assays for (**A**) miR-21-5p, (**B**) miR-18a-5p, and (**C**) miR-500a-3p on the analyzer. The bar shows ± 2 standard deviations. Each sample was measured in triplicate except for the blank sample (rep. = 5)

**Table S3** miRNA sequences used in the cross reactivity test by one-step amplification assay on the analyzer. The symbol * indicates bases identical to those of miR-21-5p. The numbers of identical bases range from 8 to 14. The highest number of consecutive identical bases is 8

**A**

**
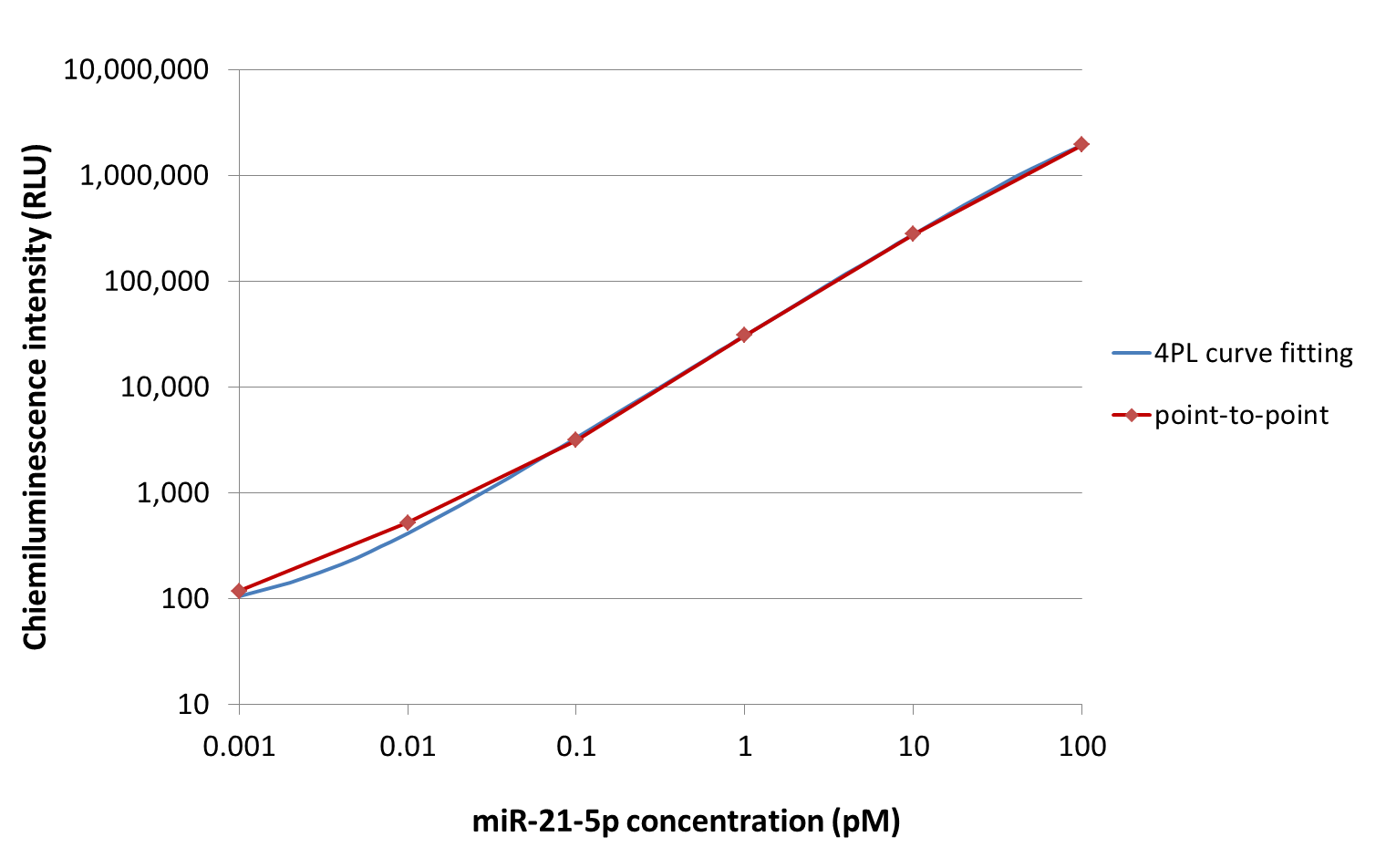
**

**B**


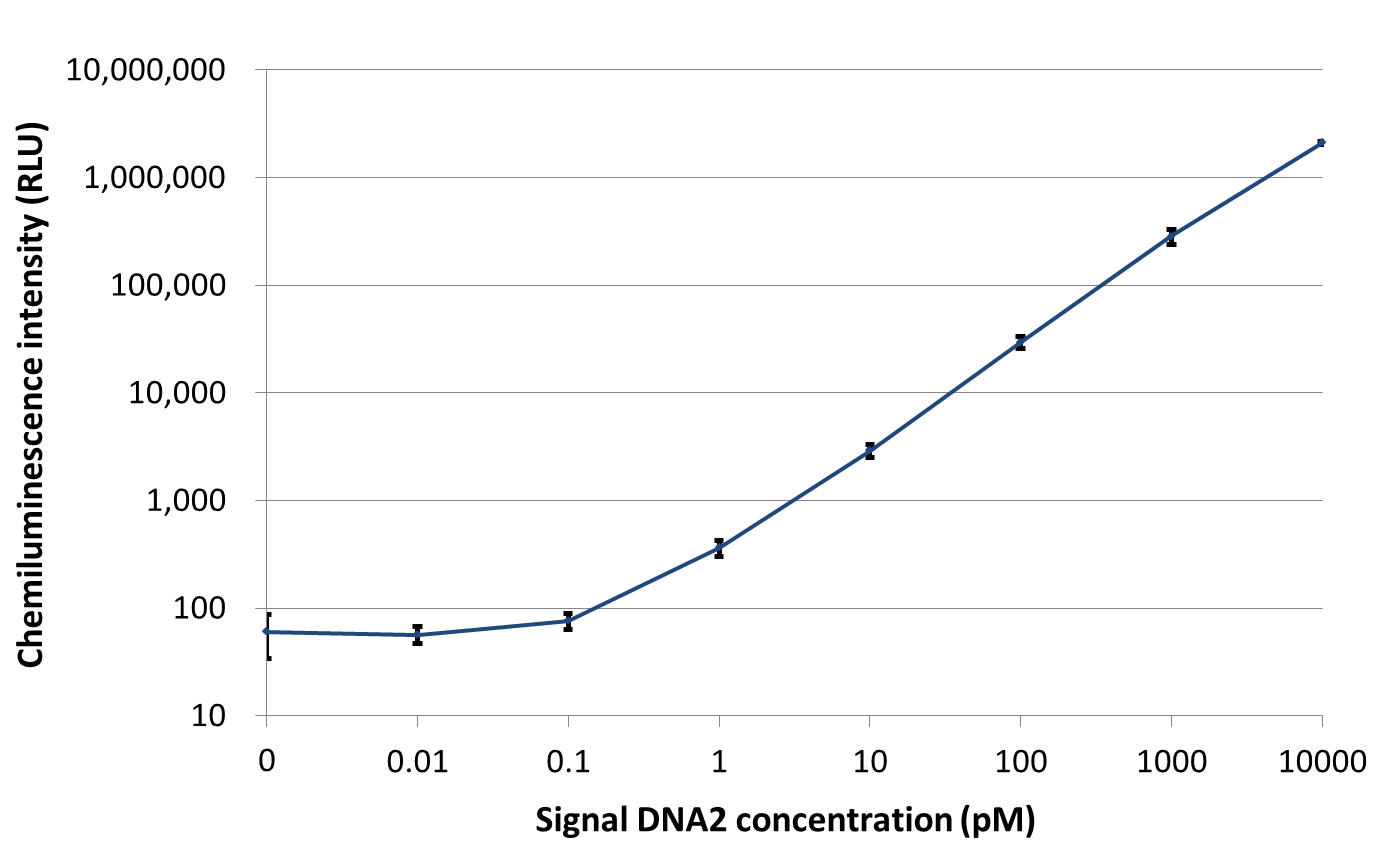


**C**


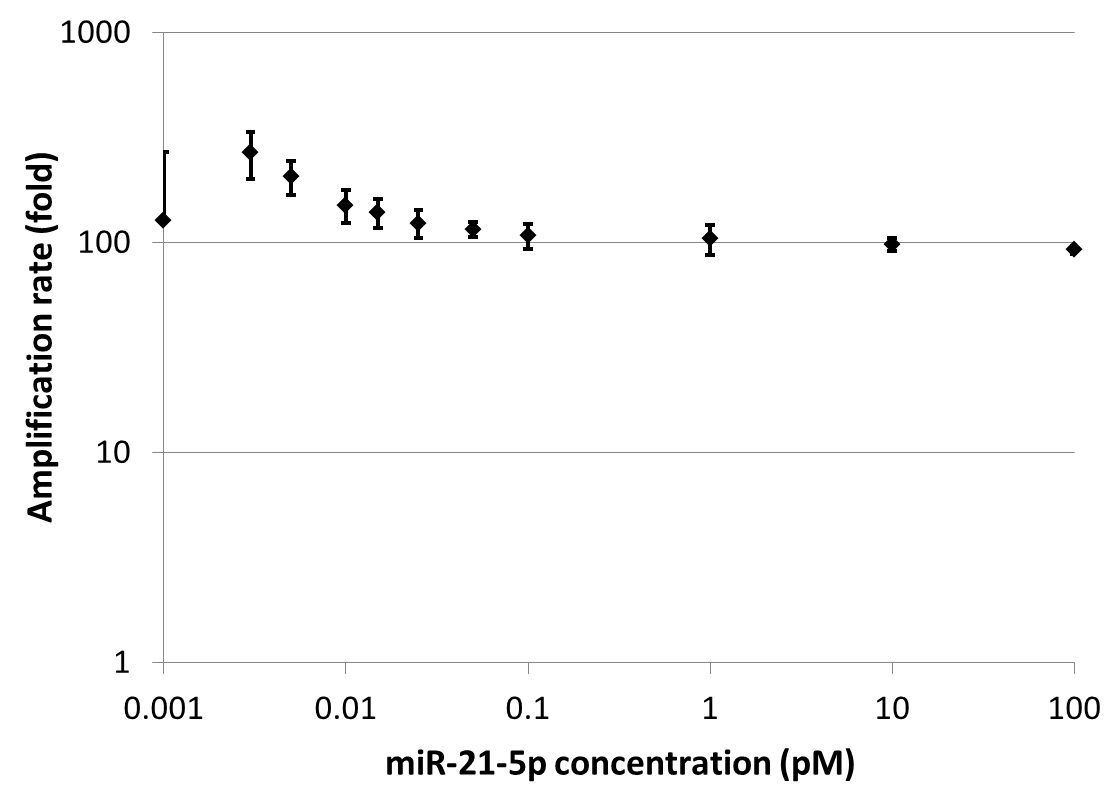


**Fig. S3** (**A**) The four parameter logistic (4PL) curve fitting of the dose response curve in the two-step amplification assay for miR-21-5p at the concentrations from 0 pM to 100 pM on the analyzer. The fitted equation was Y = 6521131 + (69.73642 – 6521131) / (1 + (X/241.1466) ^0.9764099^). The correlation coefficient was 1.0. Each sample was measured in replicates of 5.

(**B**) Calibration curve of Signal DNA2 spiked in the unreacted samples that were prepared same as the two-step amplification assay for miR-21-5p on the analyzer for calculating the amplification rates shown in Fig. S3 (C). The bar shows ± 2 standard deviations. Each sample was measured in triplicate. (**C**) Plot of amplification rates of Signal DNA2 concentrations to those of the target miR-21-5p in the two-step amplification assay for miR-21-5p on the analyzer. The bar shows ± 2 standard deviation of the amplification rate. The samples containing the target miR-21-5p at concentrations from 0.001 to 0.1 pM were measured in replicates of 20. Those at concentrations from 1 to 100 pM were measured in replicates of 5

**A**

**
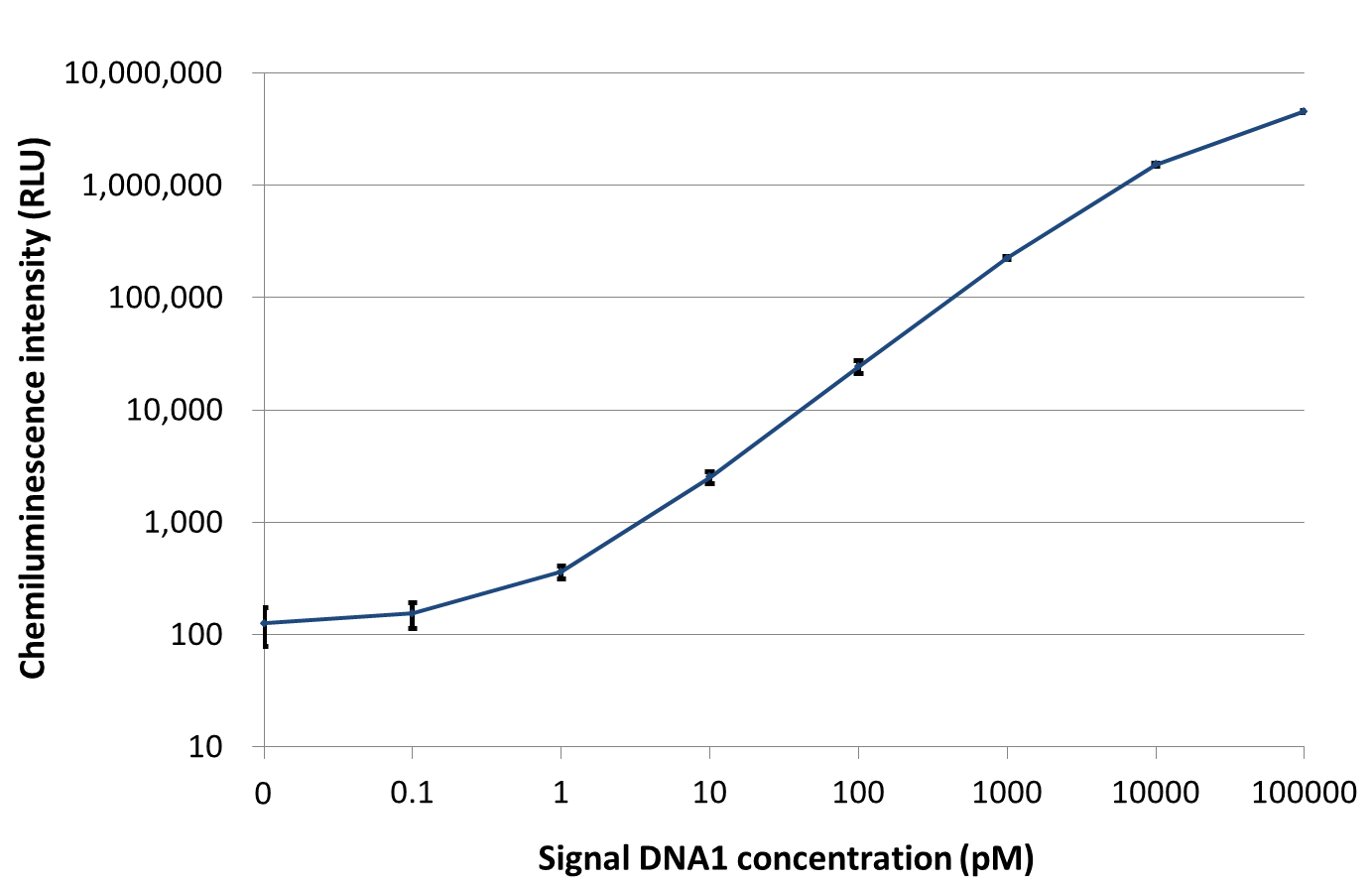
**

**B**

**
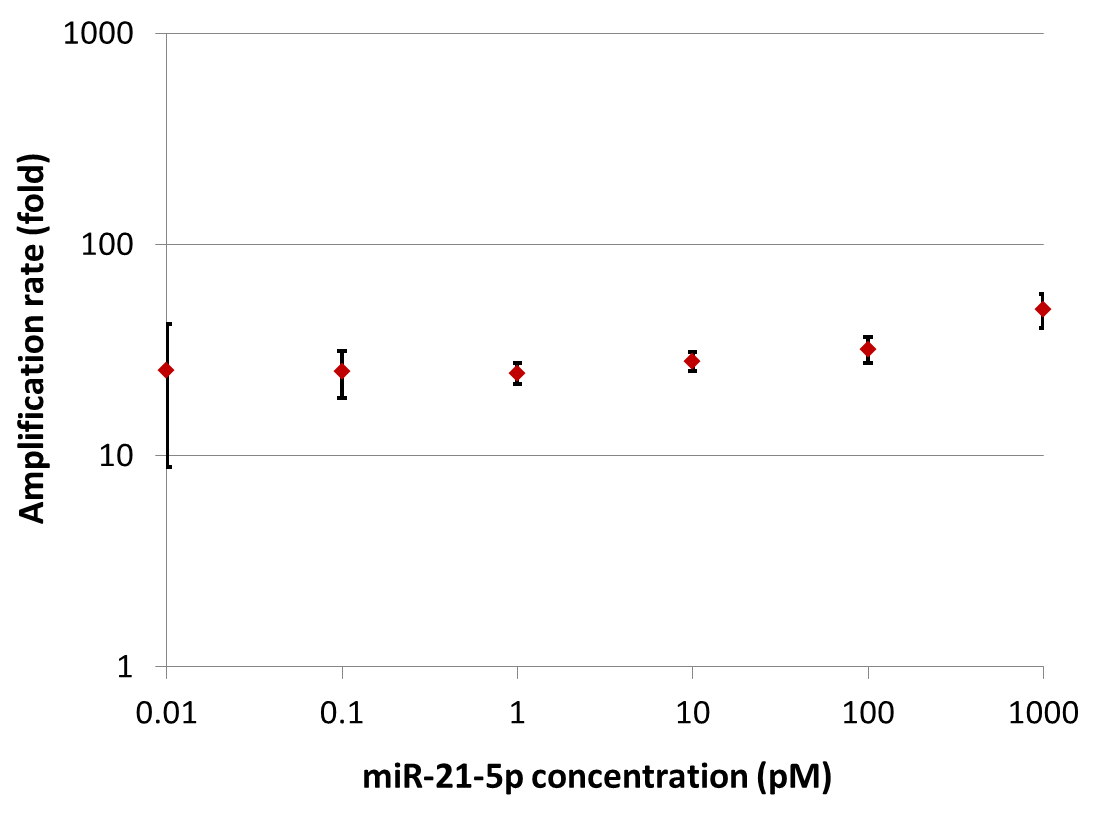
**

**Fig. S4** (**A**) Calibration curve of Signal DNA1 spiked in the unreacted samples that were prepared same as the one-step amplification assay for miR-21-5p on the analyzer for calculating the amplification rates shown in Fig. S4 (B). The bar shows ± 2 standard deviations. Each sample was measured in triplicate. (**B**) Plot of amplification rates of Signal DNA1 concentrations to those of the target miR-21-5p in the one-step amplification assay for miR-21-5p on the analyzer. The bar shows
± 2 standard deviation of the amplification rate. Each sample was measured in triplicate.

**A**

**
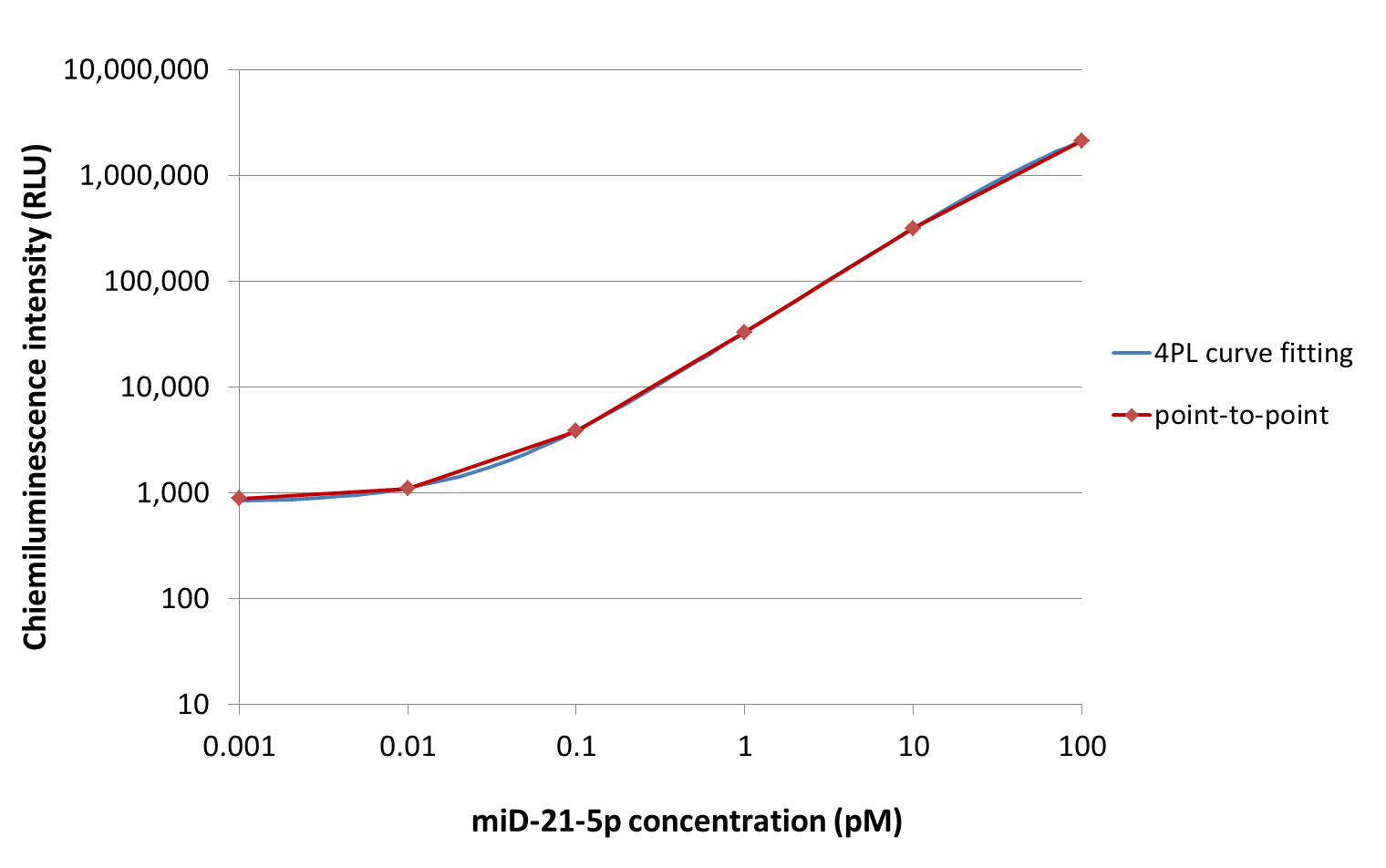
**

**B**


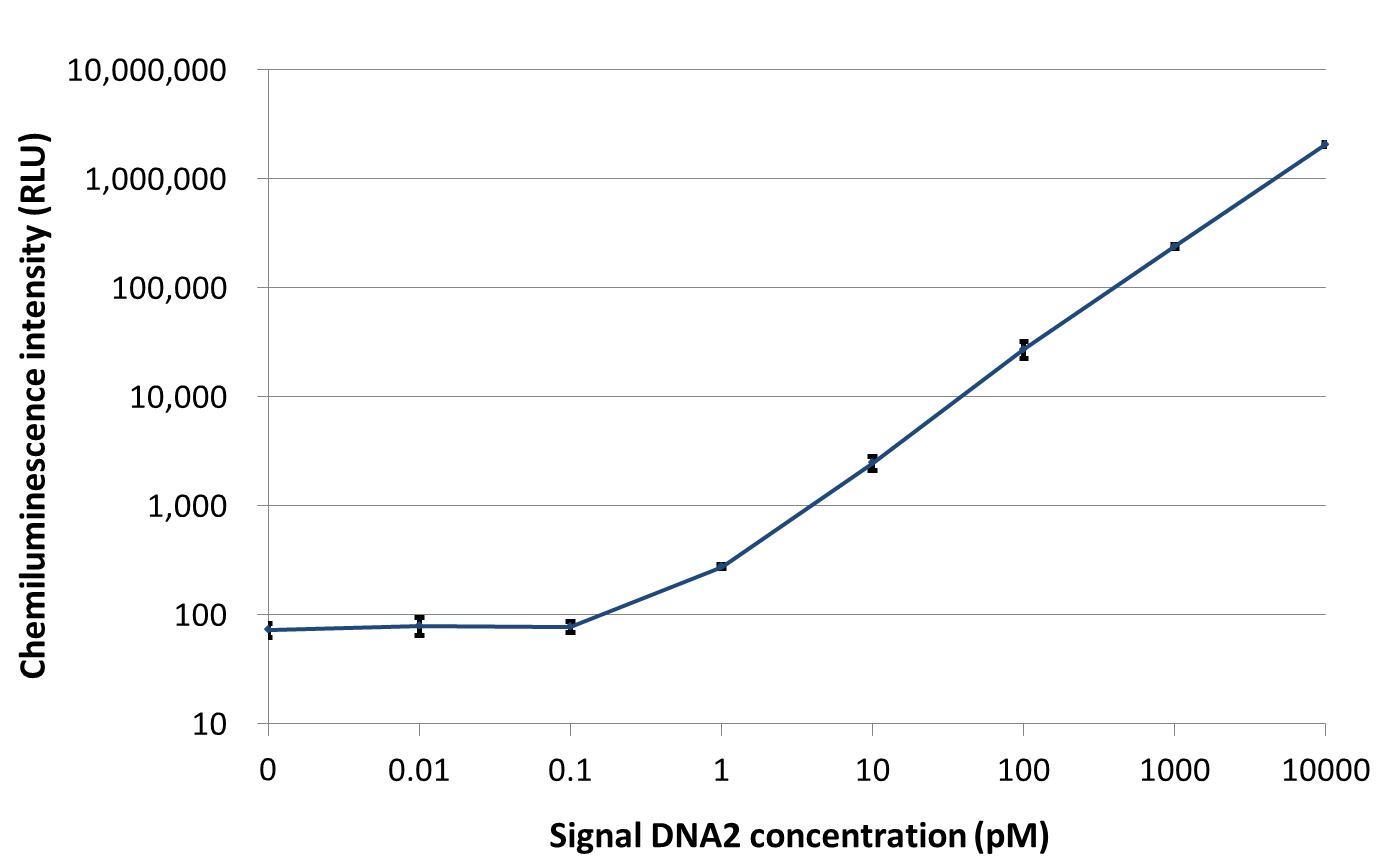


**C**


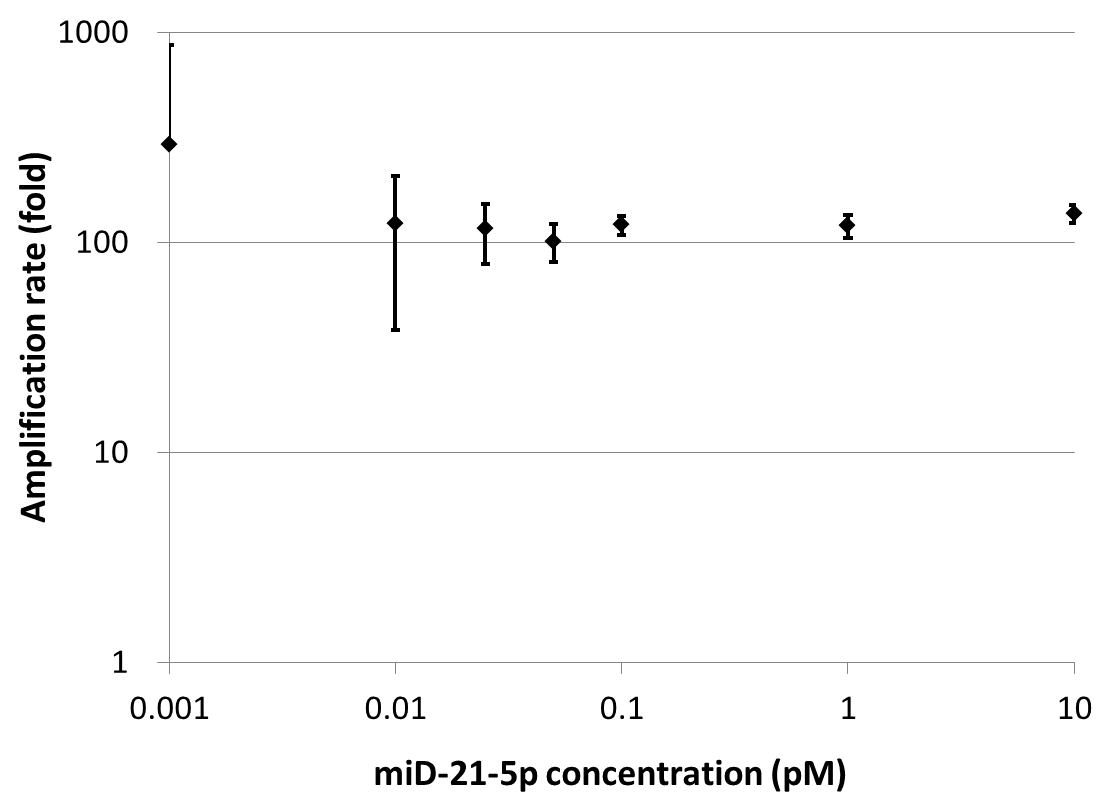


**Fig. S5** (**A**) The four parameter logistic curve fitting of the dose response curve in the two-step amplification assay for miD-21-5p at the concentrations from 0 pM to 100 pM on the analyzer. The fitted equation was Y = 5437186 + (810.5592 – 5437186) / (1 + (X/154.5906) ^1.016908^). The correlation coefficient was 1.0. Each sample was measured in replicates of 5.

(**B**) Calibration curve of Signal DNA2 spiked in the unreacted samples that were prepared same as the two-step amplification assay for miR-21-5p on the analyzer for calculating the amplification rates shown in Fig. S5 (C). The bar shows ± 2 standard deviations. Each sample was measured in triplicate. (**C**) Plot of amplification rates of Signal DNA2 concentrations to those of the target miD-21-5p in human serum in the two-step amplification assay for miR-21-5p on the analyzer. The bar shows ± 2 standard deviation of the amplification rate. Each sample was measured in replicates of 10
